# Chemerin Elicits Potent Constrictor Actions via Chemokine‐Like Receptor 1 (CMKLR1), not G‐Protein‐Coupled Receptor 1 (GPR1), in Human and Rat Vasculature

**DOI:** 10.1161/JAHA.116.004421

**Published:** 2016-10-14

**Authors:** Amanda J. Kennedy, Peiran Yang, Cai Read, Rhoda E. Kuc, Lucy Yang, Emily J. A. Taylor, Colin W. Taylor, Janet J. Maguire, Anthony P. Davenport

**Affiliations:** ^1^ Experimental Medicine and ImmunotherapeuticsLevel 6, Centre for Clinical InvestigationAddenbrooke's HospitalUniversity of CambridgeUnited Kingdom; ^2^ Department of PharmacologyUniversity of CambridgeUnited Kingdom

**Keywords:** agonist, antagonist, blood pressure, chemerin, contraction, G‐protein‐coupled receptors, human, metabolic syndrome, radioligand binding, Basic Science Research, Cell Signalling/Signal Transduction, Translational Studies, Vascular Biology

## Abstract

**Background:**

Circulating levels of chemerin are significantly higher in hypertensive patients and positively correlate with blood pressure. Chemerin activates chemokine‐like receptor 1 (CMKLR1 or ChemR23) and is proposed to activate the “orphan” G‐protein‐coupled receptor 1 (GPR1), which has been linked with hypertension. Our aim was to localize chemerin, CMKLR1, and GPR1 in the human vasculature and determine whether 1 or both of these receptors mediate vasoconstriction.

**Methods and Results:**

Using immunohistochemistry and molecular biology in conduit arteries and veins and resistance vessels, we localized chemerin to endothelium, smooth muscle, and adventitia and found that CMKLR1 and GPR1 were widely expressed in smooth muscle. C9 (chemerin149–157) contracted human saphenous vein (pD_2_=7.30±0.31) and resistance arteries (pD_2_=7.05±0.54) and increased blood pressure in rats by 9.1±1.0 mm Hg at 200 nmol. Crucially, these in vitro and in vivo vascular actions were blocked by CCX832, which we confirmed to be highly selective for CMKLR1 over GPR1. C9 inhibited cAMP accumulation in human aortic smooth muscle cells and preconstricted rat aorta, consistent with the observed vasoconstrictor action. Downstream signaling was explored further and, compared to chemerin, C9 showed a bias factor=≈5000 for the G_i_ protein pathway, suggesting that CMKLR1 exhibits biased agonism.

**Conclusions:**

Our data suggest that chemerin acts at CMKLR1, but not GPR1, to increase blood pressure. Chemerin has an established detrimental role in metabolic syndrome, and these direct vascular actions may contribute to hypertension, an additional risk factor for cardiovascular disease. This study provides proof of principle for the therapeutic potential of selective CMKLR1 antagonists.

## Introduction

Chemerin is a 16‐kDa protein that has been identified as a chemokine and adipokine with well‐established roles in inflammation and obesity.^1^ Recently, it has been reported to have a number of physiological and pathophysiological actions, suggesting a potential new role in the cardiovascular system.^2^ Chemerin enables chemotaxis of dendritic cells and macrophages to sites of injury,^3^, ^4^ alters plasma lipid and glucose levels,^5^ and promotes angiogenesis.^6^ Hypertensive patients have significantly higher plasma concentrations of chemerin compared to healthy controls, and levels of chemerin positively correlate with body mass index (BMI), systolic and diastolic blood pressure, triglycerides, and fasting plasma glucose and insulin concentrations.^7^, ^8^

Chemerin was thought to mediate its actions through a single G‐protein‐coupled receptor (GPCR) encoded by the chemokine‐like receptor 1 (CMKLR1) gene (also known as ChemR23).^6^, ^9^, ^10^, ^11^, ^12^ However, recently, chemerin has been proposed to bind and activate a second GPCR, GPR1,^13^ which has a 37% sequence identity with CMKLR1. GPR1 is currently classified as an “orphan,” although this possible pairing with chemerin has been independently confirmed.^14^, ^15^, ^16^ GPR1 was mapped to a region of interest in the British Genetics of Hypertension (BRIGHT) study,^17^ implicating it as a candidate in control of blood pressure.

To date, the actions of chemerin on vascular reactivity in humans has not been extensively studied, and a consistent pattern has not emerged from the limited studies carried out in animal models. Given that chemerin is a large protein, it is preferable to identify tool compounds to characterize its function in vitro and in vivo. Other agonists, such as the C‐terminal fragment, C9 (chemerin149–157), have been identified and are reported to mimic the actions of full‐length chemerin.^18^ Watts et al^19^ found that whereas C9 caused a concentration‐dependent contraction in a rat conduit vessel (aorta) in vitro through CMKLR1, they reported no action in vivo in this species. These researchers also found no response to chemerin in mouse aorta, whereas Kunimoto et al^20^ reported that chronic infusion of C9 caused an increase in systolic blood pressure in mice in vivo, but did not identify the receptor mediating this effect. Therefore, data from these rodent studies are currently conflicting and require clarification.

GPCRs include many of the most successfully exploited drug targets. At present, hypertension and associated pathologies remain an unresolved clinical problem, and current therapies are not cures. The chemerin signaling pathway may therefore be an additional target for translational research. We hypothesize that chemerin has significant vascular effects, in humans, mediated by 1 or both of the receptors, CMKLR1 and GPR1. In this study, we show that CMKLR1 and GPR1 are widely expressed in smooth muscle of the human vasculature. We have fully characterized a small‐molecule selective antagonist of CMKLR1 and provide compelling evidence that, in the human vasculature and rats in vivo, CMKLR1 mediates the vasoconstrictor actions of C9. We believe our data will advance the field by identifying that antagonists of CMKLR1 may be a potential new therapeutic strategy in humans.

## Methods

### Materials

Human recombinant chemerin(21–157) was purchased from R&D Systems Incorporated (Minneapolis, MN). Human C9, chemerin(149–157), was synthesised by Eurogentec (Liège, Belgium) or purchased from AnaSpec (Fremont, CA). Human C9 from AnaSpec was custom iodinated on the N‐terminal tyrosine residue ([Tyr^149^][^125^I]C9) by Anawa (Wangan, Switzerland). Human C13, chemerin(145–157), was from Phoenix Pharmaceuticals (Belmont, CA). CCX832 was kindly gifted by Dr Matt Barnes (Takeda, Cambridge, UK). All other reagents were from Sigma‐Aldrich Ltd (Poole, UK), unless otherwise stated.

### Human Tissue

Human tissues were obtained with informed consent from the Papworth Hospital Research Tissue Bank (REC reference 08/H0304/56). Experimental work was carried out with local ethical approval (REC 05/Q0104/142) and conformed to the principles outlined in the Declaration of Helsinki. Saphenous vein (SV) and mammary artery (MA) were from patients receiving coronary artery bypass grafts. Coronary artery (CA), left ventricle (LV), aorta (AO), and lung were from patients undergoing cardiac transplantation. Kidney tissue was from patients undergoing nephrectomy for nonobstructive carcinoma. Small subcutaneous resistance arteries were obtained from unused abdominal tissue and collected from patients undergoing abdominoplasty or breast reconstruction surgery. Human aortic smooth muscle cells (ASMCs) were purchased from American Tissue Culture Collection (Manassas, VA) (LGC Standards). Cells were cultured according to the supplier's instructions.

### Immunohistochemistry and Confocal Microscopy

Peroxidase/anti‐peroxidase staining was conducted, as previously described,^21^ on frozen sections of human histologically normal blood vessels, LV, lung, and kidney (all n=3) using mouse anti‐chemerin (1:300; ab72965; Abcam, Cambridge, MA); rabbit anti‐CMKLR1 (1:500; ab64881; Abcam), or rabbit anti‐GPR1 (1:300; ab150536; Abcam). To identify cellular distribution, dual‐labeling immunofluorescent staining was executed, as previously described,^21^ with frozen sections of human histologically normal blood vessels (n=3) or human ASMCs (n=3). Rabbit primary antiserum against human CMKLR1 (1:100), GPR1 (1:50), or chemerin (1:25; ab103153; Abcam) was applied together with either a mouse antihuman von‐Willebrand factor (vWF) antibody (1:50; Dako, Carpinteria, CA), or mouse antihuman smooth muscle alpha actin (SmαA; 1:100; Dako). Secondary antibodies, Alexa Fluor 633 goat antirabbit immunoglobulin G (IgG; 1:200) and Alexa Fluor 568 goat antimouse IgG (1:200; both from Life Technologies, Carlsbad, CA) were used to avoid the autofluorescence of human vessels in the 488‐nm channel.

All images were processed in Fiji,^22^, ^23^ for background subtraction, using the rolling ball method, histogram stretching, and merging of channels.

### Quantitative Polmerase Chain Reaction

RNA extraction, using Trizol Reagent and the PureLink RNA Mini Kit (Life Technologies) with DNase treatment, followed by reverse transcription with the Promega Reverse Transcription System (Promega, Madison, WI), were carried out, as in the manufacturer's instructions, on human SV (n=10), MA (n=8), CA (n=5), aorta (n=5), and ASMCs (n=6), where n is the number of different patients. cDNA products were purified using the Wizard SV Gel and polymerase chain reaction (PCR) Clean‐Up System (Promega) and subjected to real‐time quantitative PCR for 45 cycles using the ABI 7500 Real‐Time PCR System (Life Technologies) with double‐dye Taqman primer probes for human *CMKLR1* (Hs01081979_s1), *GPR1* (Hs00270990_s1), and *RARRES2* (Hs00161209_g1) and human 18S rRNA as an internal control, all from Life Technologies. Expression of *CMKLR1*,* GPR1*,* and RARRES2* (the gene for chemerin) was normalized to that of 18S. Relative expression of CMKLR1, compared to GPR1, was calculated using the ΔΔCt (delta delta threshold cycle) method and is expressed as fold‐change±SEM, that is, 2^(−ΔΔCt)^.^24^ Relative expression of RARRES2 in each tissue, compared to expression in ASMCs, was calculated using the ΔΔCt method and is expressed as fold change±SEM, that is, 2^(−ΔΔCt)^.

### Radioligand Binding Assays

Experiments were conducted in assay buffer (50 nmol/L of HEPES [pH 7.5], 1 mmol/L of CaCl_2_, 5 mmol/L of MgCl_2_, and 0.5% BSA), as previously described.^25^ Saturation binding was carried out to determine the affinity of [Tyr^149^][^125^I]C9 for either human CMKLR1 or GPR1 heterologously expressed in cells (n=3) or native receptors in human SV (n=3). Data were analyzed using Equilibrium Binding Data Analysis and LIGAND programs (KELL package; RADLIG, Cambridge, UK) to give estimates of K_D_ and B_max_ values. In subsequent experiments, homogenates were incubated with 0.1 nmol/L of [Tyr^149^][^125^I]C9 and C9 or CCX832 (0.01 nmol/L–1 μmol/L; n=3–6). Data were analyzed to obtain the dissociation constant, pK_i_ (−log_10_ dissociation constant), determined by the Cheng and Prusoff equation using the half maximal inhibitory concentration values (concentration of unlabeled ligand required to compete for 50% of the radiolabeled C9 binding) derived from the competition curves using GraphPad Prism software (version 6; GraphPad Software Inc., La Jolla, CA) (pK_i_±SEM).

### In Vitro Pharmacology of Human Vessels

To test the direct effect of chemerin on smooth muscle cells, rings (4 mm) of human SV (n=6 patients) were rubbed on the luminal surface to disrupt the endothelium and setup in organ baths, as previously described.^26^ Endothelium‐intact human resistance arteries (2‐mm rings; n=5 patients) were mounted in wire myographs to investigate the actions of chemerin on vessels physiologically relevant in blood pressure regulation.^27^ All experiments were carried out in modified Kreb's solution (89.2 mmol/L of NaCl; 29 mmol/L of NaHCO_3_; 5 mmol/L of KCl; 0.49 mmol/L of MgSO_4_.7H_2_O and 1 mmol/L of Na_2_HPO_4_.2H_2_O, 2.25 mmol/L of CaCl_2_, 5 mmol/L of fumaric acid, 5 mmol/L of glutamic acid, 10 mmol/L of glucose, and 5 mmol/L of sodium pyruvate), and vessels were maintained at 37°C with continuous oxygenation (95% O_2_/5% CO_2_). Adjacent rings were treated with either CCX832 (100 nmol/L) or vehicle (DMSO 0.001%), for 1 hour, before increasing concentrations of C9 were added. Data were analyzed (GraphPad Prism version 6; GraphPad Software Inc.) using the 4‐parameter logistic curve, to give values of pD_2_ (‐log_10_ EC_50_; EC_50_ is the concentration producing half maximal response) and E_max_ (maximum response), and antagonist affinity, pA_2_ (−logK_B_, where K_B_ is the antagonist dissociation constant), was determined using the Gaddum Schild equation. Agonist responses were subsequently normalized to the maximum response of C9 in the same tissue. All data are expressed as mean±SEM.

### Effects of C9 and CCX832 in Vivo

Animal experiments were performed according to the local ethics committee (University of Cambridge, Cambridge, CA) and Home Office guidelines under the 1986 Scientific Procedures Act.

The surgical part of the experiment was performed as previously described.^28^ Male Sprague‐Dawley rats (270±5 g) were anesthetized (3% isoflurane to induce, 1.5% isoflurane to maintain, inhaled), and level of anesthesia was monitored using the toe pinch reflex, heart rate, and respiration rate. A pressure volume catheter was inserted into the right carotid artery. Compound additions were by intravenous injection through the right jugular vein. Following baseline measurements, rats received incremental doses to determine a 70% effective concentration (EC_70_) of C9; in vivo, this dose (200‐nmol blous in 450 μL) was then used in all further studies. Using the previously published data of Kunimoto et al,^20^ which showed a chemerin‐induced increase in systolic blood pressure, compared to control, in mice in vivo to derive effect size d, a priori calculation using α=0.05 and power(1−β)=0.8 to 0.9 computed the required sample size of n=6 to 8 in each group (G*Power 3.1.9.2). We therefore stopped at 6 animals in each group because of the size of the chemerin response detected.

Rats (n=6 in each group) received 3 bolus doses (450 μL) either: C9 (200 nmol), CCX832 (40 nmol), and C9 (200 nmol)+CCX832 (40 nmol), or in control rats C9 (200 nmol), saline and C9 (200 nmol). The response to each injection was monitored until returning to baseline or for at least 10 minutes. Rats were euthanized by exsanguination under high isoflurane (5%) followed by cervical dislocation. Data analysis was performed using LabChart 8, as previously described.^29^ Peak effects of C9 in the absence and presence of CCX832, saline, and CCX832 on mean arterial blood pressure (MAP) and heart rate (HR) are expressed as a change from baseline and tested for significance with a Student *t* test using using GraphPad Prism software (version 6; GraphPad Software Inc.). To determine the effect of CCX832 on C9 response, within each rat, the second repeated dose of C9 in the absence and presence of CCX832 was expressed as a percentage of the first dose of C9 and tested for significance with a Student *t* test using using GraphPad Prism (version 6; GraphPad Software Inc.).

### Rat Aorta Contraction Assay

Rings (4 mm) of endothelium‐denuded rat aorta (n=4 rats) were set up in organ baths as described for human vessels, and responses to increasing concentration of C9 were measured. To investigate the role of cAMP in the constrictor response, the following protocol was adapted from Alsaqati et al.^30^ Vessels were preconstricted with U46619 (100 nmol/L) and relaxed back to baseline with NKH477 (300 nmol/L), which directly activates adenylyl cyclase, before responses to C9 were measured. Responses are expressed as a percentage of the maximum response to 100 mmol/L of KCl added at the end of each experiment. Data were analyzed using a 4‐parameter logistic curve (GraphPad Prism 6; GraphPad Software Inc.), to give values of pD_2_ and E_max_. All data are expressed as mean±SEM.

### cAMP Assays of Human ASMCs

Confluent cultures of human ASMCs (n=3) labeled with [2,8‐^3^H] adenine (1 μCi/mL) were treated with CCX832 (100 nmol/L) or vehicle (DMSO 0.001%) before addition of NKH477 (3 μmol/L) in the absence or presence of C9 (1 μmol/L). Incubations were terminated by aspirating the medium and addition of ice‐cold trichloroacetic acid (5% v/v). ^3^H‐labeled adenine nucleotides were separated as reported previously.^31^ Accumulation of ^3^H‐cAMP is expressed as a fraction of the sum of ^3^H‐ATP, ^3^H‐ADP, and ^3^H‐cAMP activities. Results with chemerin‐related ligands were then expressed as a percentage of the response evoked by NHK477 alone and presented as mean±SEM.

### Cell‐Based Functional Assays

β‐Arrestin recruitment (CMKLR1 and GPR1) and cAMP assays (CMKLR1; DiscoverX, Fremont, CA) were carried out with CHO‐K1 cells expressing either human CMKLR1 or GPR1, according to the manufacturer's instructions. In both assays, agonist concentration‐response curves, measured in relative light units, were fitted to a 4‐parameter logistic concentration response curve in GraphPad Prism software (version 6; GraphPad Software Inc.), and values of pD_2_ and E_max_ were calculated. Data were subsequently normalized to the maximum response to C13 used as the reference agonists in each assay. For experiments with CCX832, antagonist affinities, pA_2_, were determined using the Gaddum Schild equation in GraphPad Prism (version 6; GraphPad Software Inc.).

### Analysis of C9 and C13 Signalling Pathway Bias at CMKLR1

In cell‐based assays (DiscoverX), where chemerin, C9, and C13 were all tested in the same experiment, data were analyzed as previously described^32^ to define the pathway signaling profile of C‐terminal fragments C9 and C13 compared with chemerin and to quantify any bias for the individual signaling pathways investigated.

### Statistical Analysis

All results are reported as mean±SEM. For human ASMC studies in humans and in vivo animal studies, a 2‐tailed Student *t* test was used, as appropriate. Statistical significance was taken at the 5% level.

## Results

### Chemerin, CMKLR1, and GPR1 are Present in the Human Cardiovascular System

**Figure 1:**
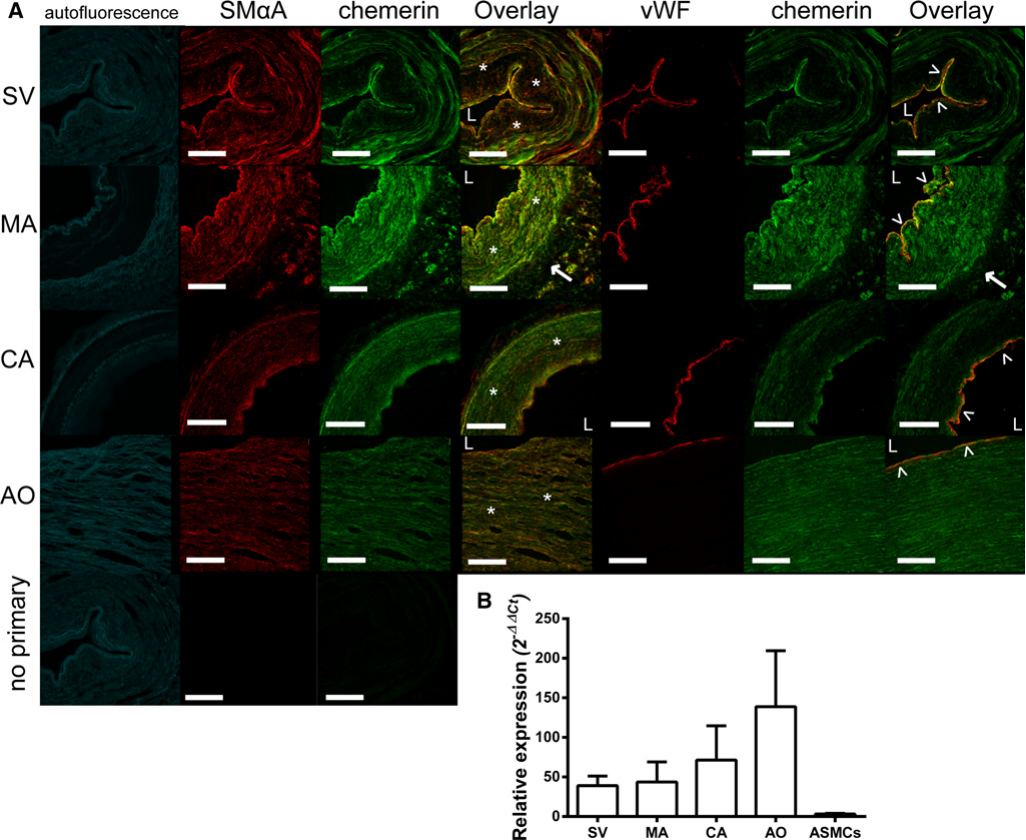
Chemerin expression in human vessels: Representative photomicrographs show chemerin (green) in smooth muscle cells (*) and endothelium (˄) of human (A) saphenous vein (SV), mammary artery (MA), coronary artery (CA), and aorta (AO; all n=3 patients), with cell markers (red) smooth muscle alpha actin (SMαA) or von Willebrand factor (vWF) and autofluorescence in the 488‐nm channel (in blue). L denotes the lumen of the vessel and → expression in the adventitial layer of the MA. Scale bars=500 μm. B, Expression of mRNA levels of chemerin in human vessels and ASMCs are shown relative to expression in ASMCs and expressed as mean±SEM (n=5–10 patients). ASMCs indicates aortic smooth muscle cells.

**Figure 2:**
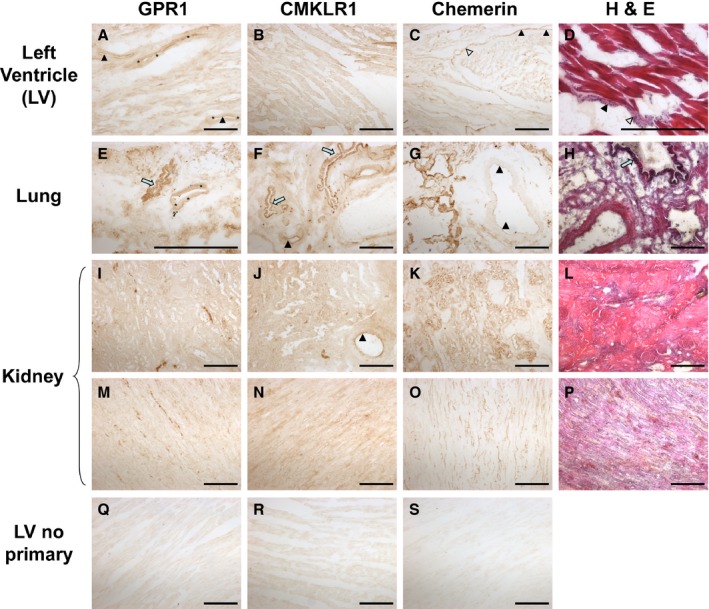
Representative photomicrographs of human left ventricle (LV), lung, and kidney show: GPR1, CMKLR1 and chemerin in human cardiomyocytes (A, B, and C, respectively); pulmonary vessels and bronchioles (E, F, and G, respectively); renal tubular epithelial cells (I, J, and K, respectively); renal medulla rays (M and N) and renal capillaries (O); and the corresponding no primary antibody controls (Q, R, and S). Triangular arrows indicate endothelial lining, and asterisks indicate smooth muscle layer. Blue arrows indicate bronchioles. Positive staining is shown as a brown product under the light microscope. H&E staining was carried out on the same tissues (D, H, L, and P) with hematoxylin staining nuclei (blue) and eosin staining protein structures (red). Scale bars=500 μm. CMKLR1 indicates chemokine‐like receptor 1; GPR1, G‐protein‐coupled receptor 1; H&E, hematoxylin and eosin.

mRNA encoding chemerin, CMKLR1, and GPR1 and the corresponding proteins were widely expressed in human large conduit arteries and veins and in resistance vessels. Chemerin was present in the endothelium and smooth muscle medial layer, as defined by vWF and SMαA staining, respectively, of histologically normal human SV, MA, AO, and CA, as well as the adventitial layer of MA (Figure 1A). In sections of human LV, lung, and kidney, chemerin was localized to endothelial cells of small blood vessels and to endocardial endothelial cells, epithelial cells of the lung bronchioles, and tubules and endothelial cells in the kidney (Figure 2). Chemerin mRNA levels were similar in human SV, MA, CA, and AO and less abundant in ASMCs (Figure 1B).

**Figure 3:**
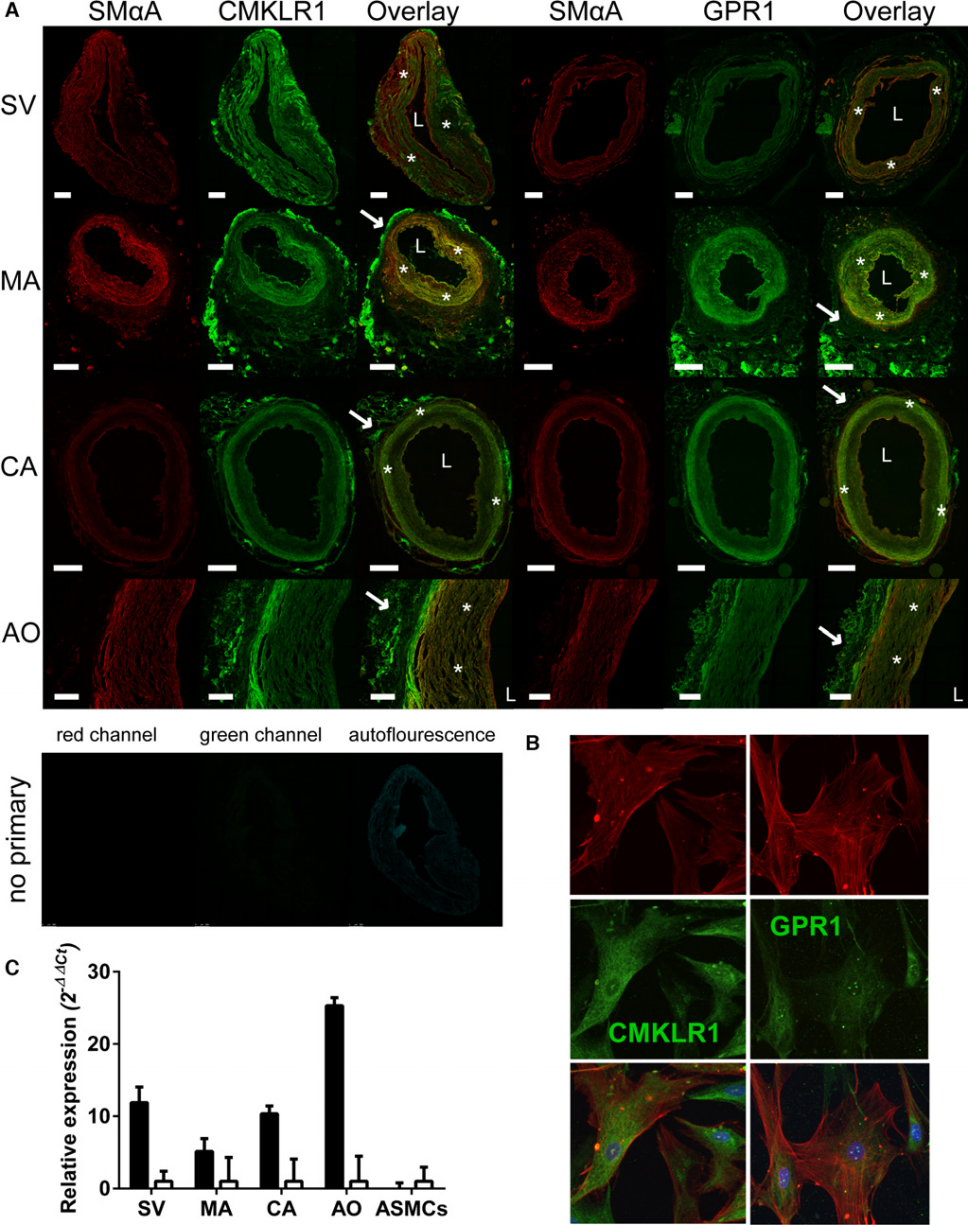
CMKLR1 and GPR1 expression in human vessels: Representative photomicrographs show CMKLR1 and GPR1 (green) in smooth muscle cells (*) and adventitia (→) of human (A) saphenous vein (SV), mammary artery (MA), coronary artery (CA), and aorta (AO) and (B) ASMCs (all n=3 patients) with cell marker (red) smooth muscle alpha actin (SMαA) and a corresponding no primary control, including autofluorescence, seen in the 488‐nm channel. L denotes the lumen of the vessel. Cell nuclei stained with 4’,6‐diamidino‐2‐phenylindole (blue). Scale bars=500 μm. C, Expression of mRNA for CMKLR1 (black) and GPR1 (open bars) in human vessels and ASMCs are shown relative to expression of GPR1 and expressed as mean±SEM (n=5–10 patients). ASMCs indicates aortic smooth muscle cells; CMKLR1, chemokine‐like receptor 1; GPR1, G‐protein‐coupled receptor 1.

CMKLR1 and GPR1 were detected in smooth muscle of resistance vessels in human LV, lung, and kidney sections and, additionally, to cardiomyocytes, bronchioles, and renal tubules (Figure 2). CMKLR1 and GPR1 localized to the medial layer of healthy human vessels (Figure 3A) and were expressed in human ASMCs (Figure 3B). Both receptors were also present in the adventitial layer of adipose tissue surrounding human MA, CA, and AO (Figure 3A). Analysis of mRNA for each receptor revealed that CMKLR1 was expressed at higher levels than GPR1 in human vessels, but there was relatively more GPR1 mRNA in human ASMCs (Figure 3C).

### Activation of CMKLR1 Caused Contraction of Human Vessels

**Figure 4:**
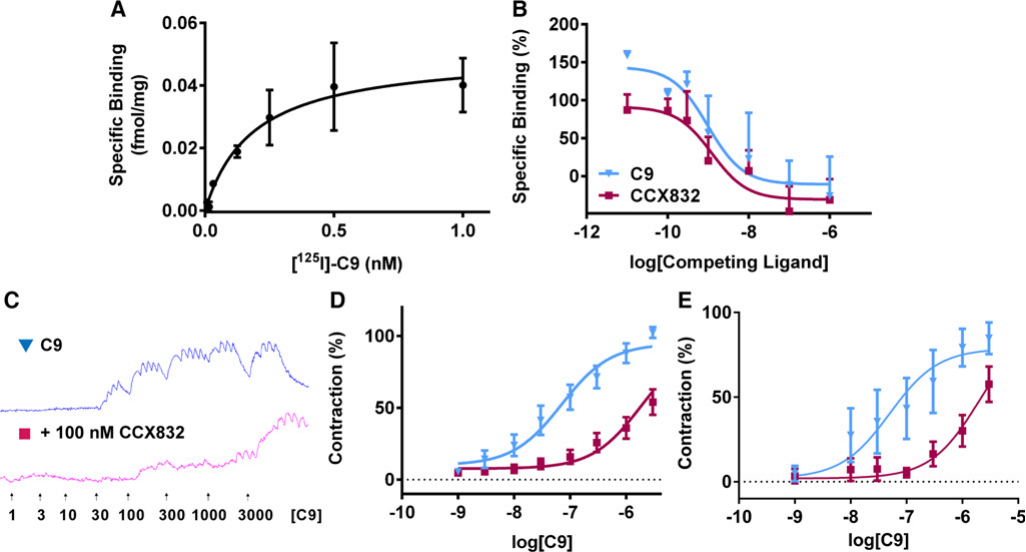
Binding and function of CMKLR1 in human tissues. Radiolabeled binding studies reveal that (A) C9 binds to 1 site, with a subnanomolar affinity (K_D_ = 0.53±0.31 nmol/L; B_max_=0.05±0.007 fmol/mg; n=3), and B, unlabeled C9 (▼, pK_i_=9.11±0.52; n=5) and CCX832 (■, pKi=8.65±0.38; n=6, pink) compete for all the binding of radiolabeled C9 at native receptors. C9 induced a concentration‐dependent contraction of human vessels (blue), which was blocked by pretreatment with CCX832 (100 nmol/L, pink). Raw trace of the response in SV (C) and the corresponding concentration‐response curves to (▼) C9 and (■) C9+100 nmol/L CCX832 in human SV (D; n=6 patients) and resistance arteries (E; (n=5 patients). Responses are shown as % of maximal response to C9 and expressed as mean±SEM. CMKLR1 indicates chemokine‐like receptor 1; SV, saphenous vein.

Radiolabeled binding studies with [Tyr^149^][^125^I]C9, in human SV homogenate, revealed that C9 binds to a single site, with a subnanomolar affinity of K_D_=0.53±0.31 nmol/L and B_max_=0.05±0.007 fmol/mg (Figure 4A). Unlabeled C9 (pK_i_=9.11±0.52) and reported CMKLR1 antagonist, CCX832 (pKi=8.65±0.38), compete for all the binding of radiolabeled C9 (Figure 4B), suggesting that C9 is binding only to native CMKLR1 receptors in human SV.

C9 caused a concentration‐dependent contraction of human SV (pD_2_=7.30±0.31). Preincubating the SV with 100 nmol/L of CCX832 before adding C9 caused a rightward shift of the curve (pD_2_=6.57±0.62; Figure 4C and 4D) with a pA_2_=8.39±0.15. Similarly, in the more physiologically relevant human resistance arteries, C9 caused a concentration‐dependent contraction (pD_2_=7.05±0.54), which was antagonized when the vessel was pretreated with CCX832, (pA_2_=7.96±0.34; Figure 4E). This study shows, for the first time, that activation of CMKLR1 causes contraction of human blood vessels.

### CMKLR1 Is a Novel Regulator of Blood Pressure in Rats In Vivo

**Figure 5:**
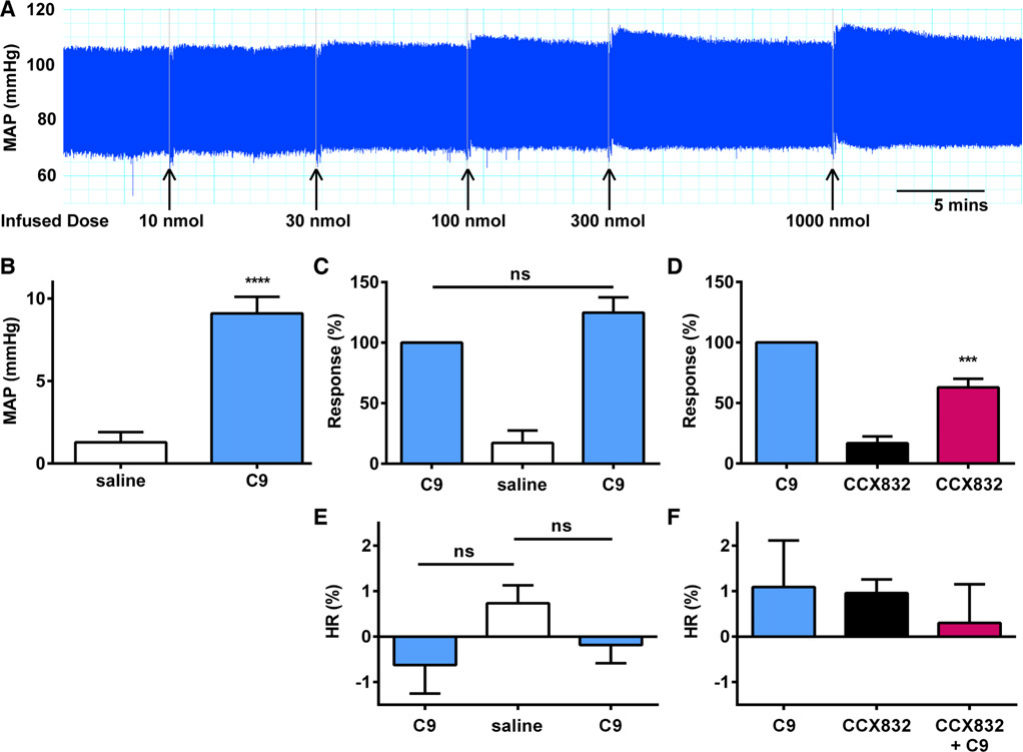
C9 is active in vivo in rats. A, Example trace of initial DRC to C9 carried out to determine EC_70_ value of 200 nmol. B, C9 (200 nmol, blue) caused a significant (Student *t* test; ****P<0.0001; n=12 rats) increase in mean arterial blood pressure (MAP) in vivo in normotensive rats. C, Repeated doses of C9 (200 nmol) caused similar increases in blood pressure (n=6 rats). D, CCX832 alone (40 nmol, black) had no further effect on BP than saline control (white); however, when infused with C9 (pink), there was a significant (Student *t* test; ***P<0.001; n=6 rats) reduction in pressor response. Responses are shown as absolute values (A and B) or expressed as % of first C9 dose (C and D). E, Effect of C9 (200 nmol, blue) on heart rate (HR) in vivo compared to saline controls (open bars). F, Effect of CCX832 alone (40 nmol, black) and C9 in the presence of CCX832 (pink) on HR. Data are expressed as the % difference in HR of peak response compared to baseline, mean±SEM. BP indicates blood pressure; DRC, dose‐response curve; EC_70_, 70% effective concentration.

C9 caused a dose‐dependent increase in MAP in rats (Figure 5A), in vivo, with an EC_70_ of 200 nmol. C9 (200 nmol) caused a significant increase in MAP of 9.1±1.0 mm Hg (Figure 5B). Repeated doses of C9 gave reproducible increases in MAP (Figure 5C), with no evidence of desensitization. CCX832 alone had no effect on MAP; however, infusion of CCX832 with C9 caused a significant reduction in response compared to C9 alone (Figure 5D). C9 and CCX832 had no effect on heart rate compared to saline control (Figure 5E and 5F). Therefore, these data identify CMKLR1 as a novel regulator of blood pressure in vivo.

### Chemerin Caused Vasoconstriction Through Inhibition of cAMP Accumulation

**Figure 6:**
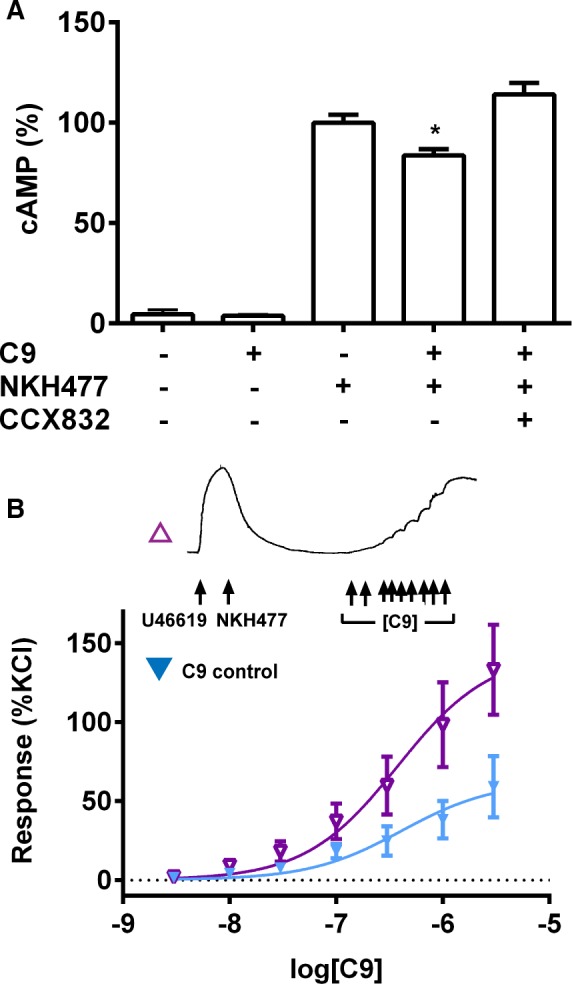
Inhibition of cAMP accumulation by C9 through CMKLR1. A, In human ASMCs stimulated with NKH477 (3 μmol/L), C9 (1 μmol/L) significantly (Student *t* test; *P<0.05; n=3 patient cell lines) inhibited cAMP accumulation. Pretreatment with CCX832 (100 nmol/L) blocked this response. Responses are expressed as mean±SEM. (B) In rat aorta primed to produce cAMP (see trace), C9 induced greater contractions (▼), compared to a C9 control response from baseline (▼). Responses are shown as % of contraction evoked by 100 mmol/L of KCl and expressed as mean±SEM (n=4 rats). ASMCs indicates aortic smooth muscle cells; CMKLR1, chemokine‐like receptor 1.

In human ASMCs stimulated with NKH477 to directly activate adenylyl cyclase, addition of C9 caused a significant reduction in the amount of cAMP accumulated. This inhibition of cAMP accumulation by C9 was blocked by CCX832 (Figure 6A), suggesting that C9 is modulating the response through CMKLR1. In preconstricted rat aorta where NKH477 had caused relaxation to the basal state, C9 induced more contraction (pD_2_=6.42±0.17; E_max_=144±17% of that evoked by KCl) than it did in naïve tissue (pD_2_=6.39±0.22; E_max_=62±10%; Figure 6B). Consistent with a role in contraction, these mechanistic studies show that activation of CMKLR1 in the vasculature inhibits cAMP accumulation.

### Confirmation of the Pharmacology of C9 and Antagonist CCX832 at Chemerin Receptors CMKLR1 and GPR1

To characterize the binding and activation of CMKLR1 and GPR1, the human receptors were individually expressed in CHO‐K1 cells. Radiolabeled C9 bound to CMKLR1 and GPR1 with a similar subnanomolar affinity (K_D_=0.28±0.07 and 0.87±0.23 nmol/L, respectively).

**Figure 7:**
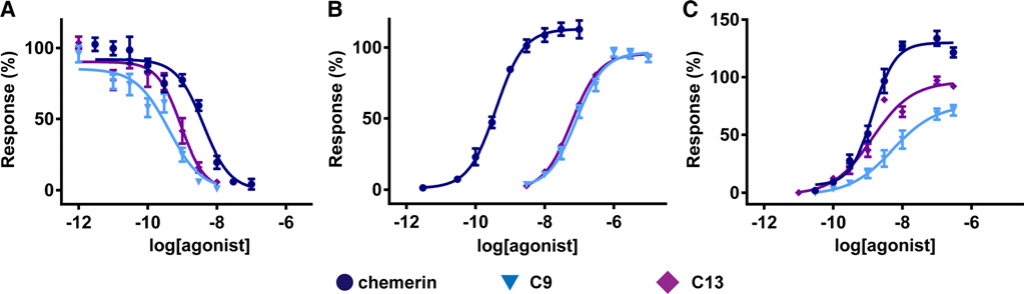
Functional activity of (●) chemerin, (▼) C9 and (♦) C13 at heterologously expressed CMKLR1 and GPR1. Potencies of peptides were determined from measurements of inhibition of cAMP accumulation through CMKLR1 (A) and β‐arrestin recruitment through CMKLR1 (B) and GPR1 (C). Responses are shown as % of the maximal response to C13 and show mean±SEM (n=3–9 independent replicates). Calculated functional parameters are presented in Table 1. CMKLR1 indicates chemokine‐like receptor 1; GPR1, G‐protein‐coupled receptor 1.

Activities of full‐length chemerin and C9 were compared in downstream signaling assays. The C‐terminal fragment C13 was included to further investigate a trend of biased signaling. In cell‐based functional assays with CMKLR1, chemerin, C9, and C13 exhibited comparable maximum responses (Table 1). C‐terminal fragments, C9 and C13 (pD_2_=9.39±0.09 and 9.12±0.12, respectively) were more potent than chemerin (pD_2_=8.45±0.10) at inhibiting forskolin‐evoked cAMP accumulation (Figure 7A). However, compared to chemerin (pD_2_=9.37±0.05), C9 and C13 were less potent (pD_2_=7.09±0.06, and 7.15±0.04, respectively) at recruiting β‐arrestin (Figure 7B). Using chemerin as the reference endogenous ligand, bias analysis confirmed that the C‐terminal fragments had a bias factor of ≈3000 to 5000 for inhibition of cAMP accumulation compared to β‐arrestin recruitment (Table 2).

**Table 1:** Pharmacological Data for Chemerin Peptides at CMKLR1 and GPR1 as Observed in Radioligand Binding and Functional Assays (Figure 7)

		CMKLR1	GPR1
Ligand binding	[^125^I]‐C9	K_D_=0.28±0.07 nmol/L (n=3)	K_D_=0.87±0.23 nmol/L (n=3)
Competition binding	C9	pKi=9.21±0.14 (n=3)	pK_i_=8.81±0.17 (n=3)
	CCX832	pK_i_=9.16±0.42 (n=6)	No effect (n=3)

		pD_2_	E_max_ (%)	n	pD_2_	E_max_ (%)	n
β‐Arrestin recruitment	Chemerin	9.37±0.05	113±6	3	9.05±0.09	134±6	3
	C9	7.09±0.06	96±4	3	8.09±0.16	71±5	7
	C13	7.15±0.04	94±2	6	8.65±0.14	97±3	9
cAMP inhibition	Chemerin	8.45±0.10	4.3±3.7	3	—		
	C9	9.39±0.09	0.9±0.4	4	—		
	C13	9.12±0.12	5.7±1.5	3	—		

CMKLR1 indicates chemokine‐like receptor 1; GPR1, G‐protein‐coupled receptor 1.

**Table 2:** C‐Terminal Fragments Display Bias Towards G_i_ Pathway over β‐Arrestin Recruitment, Compared to Chemerin at CMKLR1

	β‐Arrestin Assay			cAMP Assay			cAMP vs β‐Arrestin	
	logR^a^	ΔlogR	RE^b^	logR^a^	ΔlogR	RE^b^	ΔΔlogR	Bias Factor
Chemerin	9.58±0.04	0.00±0.06	1	8.38±0.04	0.00±0.06	1	0.00±0.09	1
C13	7.20±0.03	−2.37±0.04	0.004	9.51±0.14	1.14±0.20	13.8	3.52±0.21	3278.44
C9	6.74±0.04	−2.83±0.05	0.001	9.27±0.06	0.89±0.08	7.73	3.72±0.10	5280.40

CMKLR1 indicates chemokine‐like receptor 1.

a logR=log_10_(Ƭ/K_A_) where Ƭ is a measure of agonist efficacy and K_A_ is the agonist functional affinity.

b RE is the relative effectiveness compared to chemerin.

Chemerin was functionally active at the GPR1 receptor in β‐arrestin recruitment assays (pD_2_=9.05±0.09; Figure 7C). C9 and C13 (pD_2_=8.09±0.16 and 8.65±0.14, respectively) were less potent at recruiting β‐arrestin and appear to be partial agonists (E_max_=71±5% and 97±3%, respectively) compared to chemerin (E_max_=134±6%).

**Figure 8:**
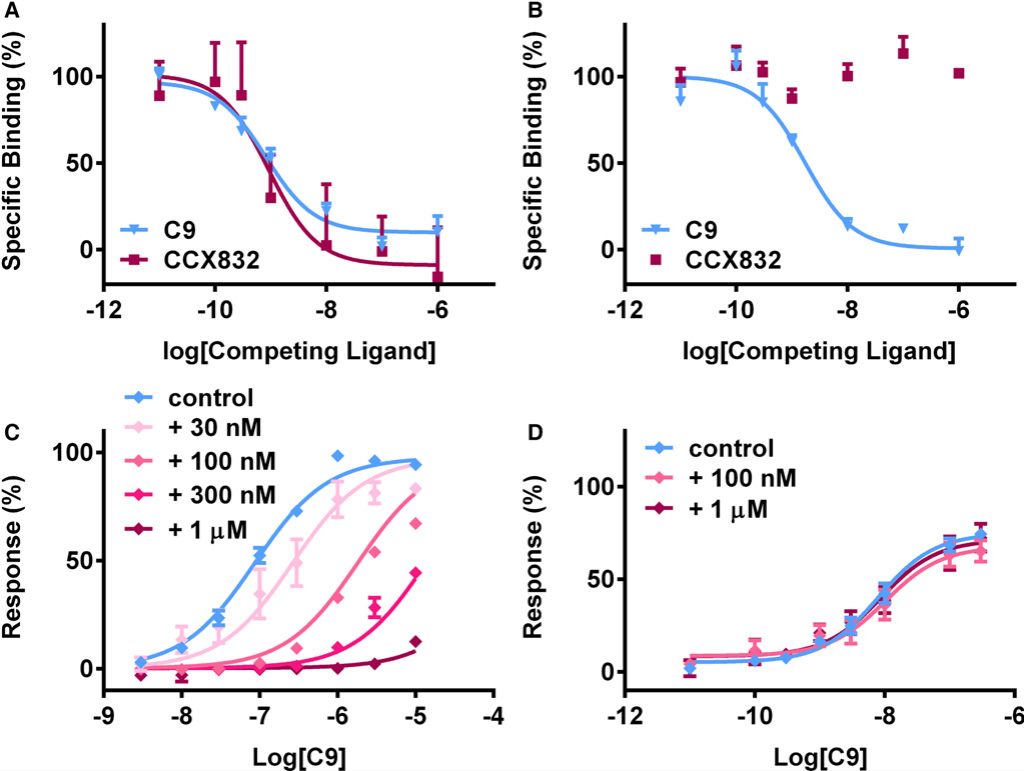
Characterization of small‐molecule CCX832 using cells expressing CMKLR1 or GPR1. Radiolabeled competition binding studies, with CCX832 (■) and unlabeled C9 (▼) as the competing ligands, revealed that CCX832 competed for all binding of radiolabeled C9 to CMKLR1 (A), but had no effect on binding to GPR1 (B). Data are expressed as a % of the total specific binding and show mean±SEM (n=3–6 independent replicates). pK_i_ values are shown in Table 1. In β‐arrestin recruitment assays, increasing concentrations of CCX832 caused a rightward shift of C9 response at CMKLR1 (C) (pA_2_=8.32±0.04), but had no effect at GPR1 (D). Data are expressed as % of the maximum response to C13 in each assay and show mean±SEM (n=3 independent replicates). CMKLR1 indicates chemokine‐like receptor 1; GPR1, G‐protein‐coupled receptor 1.

To confirm the selectivity of reported CMKLR1 antagonist, binding and functional studies were carried out using the same cell lines. CCX832 competed for radiolabeled C9 binding to CMKLR1 (pK_i_=9.16±0.42; Figure 8A), but not GPR1 (Figure 8B). It blocked the C9‐mediated β‐arrestin recruitment at CMKLR1 (pA_2_=8.32±0.04, Figure 8C), but had no effect on the C9 response at GPR1 (Figure 8D).

## Discussion

This is the first study to investigate fully the role of chemerin on human vascular contractility. We have identified that a single receptor, CMKLR1, mediates the vasoconstrictor actions of C9 in humans and, importantly, confirmed that the actions of C9 and CMKLR1 selective antagonist, CCX832, translate into the rat in vivo, identifying a robust animal model required for preclinical drug development studies.

### Chemerin and Chemerin Receptors Are Widely Expressed in the Human Vasculature

Localization of GPR1 has not been investigated in the human cardiovascular system before, and expression of chemerin and CMKLR1 has only been identified in atherosclerotic coronary arteries and aorta^33^ and isolated mesenteric arteries.^19^ Before all else, it was therefore imperative to definitively locate the 3 potential components of the chemerin axis in healthy human cardiovascular tissues to begin to understand their function.

Our results show that mRNA and protein of both chemerin receptors, CMKLR1 and GPR1, are widely expressed in smooth muscle, the contractile element, of large conduit vessels and small internal vessels within organs in the human cardiovascular system, suggesting that they could have an important role in modulation of vascular tone. This is consistent with previous findings of CMKLR1 expression on smooth muscle in human^33^ and rat^19^ vessels. There was relatively more CMKLR1 mRNA than GPR1 mRNA in all human vessels tested, although GPR1 mRNA was more abundant than CMKLR1 mRNA in human ASMCs. The difference may be a consequence of culturing ASMCs or reflect that homogenates contained smooth muscle, endothelial cells, and adventitia. CMKLR1 was previously reported to be expressed on cultured endothelial cells,^6^ and GPR1 has not.

Chemerin mRNA and protein localized to the endothelium, smooth muscle, and fat cells of human vessels, placing it in close proximity to its receptors, suggesting that it could act as a locally released mediator and levels could be elevated in pathologies, such as obesity and metabolic syndrome. This staining is consistent with other literature, which found that chemerin was expressed in dermal microvascular endothelial cells^34^ and the smooth muscle medial layer of arteries.^33^ Interestingly, expression in smooth muscle is similar to another adipokine, adiponetin.^35^ Chemerin is released as biologically inactive prochemerin and must be cleaved at the C‐terminus for activation, by enzymes present in the vasculature.^36^, ^37^, ^38^ Our chemerin antibody was designed to detect expression of all chemerin isoforms, and so the identity of active chemerin proteins or peptides within the human vessel wall has not yet been determined.

### CCX832 Is a Highly Selective Antagonist for CMKLR1

Chemerin is proposed to act through 2 GPCRs.^9^, ^10^, ^13^ Our results are in agreement with 2 previous studies: Chemerin activates both CMKLR1 and GPR1 with a similar potency in β‐arrestin recruitment assays.^16^ At both receptors, radiolabeled C9 bound with a similar subnanomolar affinity,^13^ and the C‐terminal fragments stimulated β‐arrestin recruitment. This confirms the pairing of GPR1 with the ligand chemerin and shows that both CMKLR1 and GPR1 are activated by C9.

Given that there are two chemerin receptors, and both are present in human vessels, it is crucial to determine whether 1 or both receptors mediate the effect of chemerin, if these findings are going to translate into a clinical setting. CCX832 is a small‐molecule compound, previously only characterized by high‐throughput screen.^19^ Since then, GPR1 has been identified as a chemerin receptor because of its high sequence identity with CMKLR1,^13^ and our data confirm this pairing. We present the full characterization of CCX832, through binding and functional studies, to confirm that it is selective for CMKLR1 and has no effect at GPR1.

### Novel Role of CMKLR1 in Blood Pressure Regulation

We present, for the first time, compelling evidence that CMKLR1 mediates the vascular actions of chemerin in human. Our data show that C9 caused a concentration‐dependent contraction of human SV, which was blocked by CCX832, consistent with the radiolabeled binding data that C9 binds and functions through CMKLR1. This effect of C9 was not limited to the venous system, but, importantly, it was also observed in the more physiologically relevant human resistance arteries, which directly contribute to blood pressure. In human ASMCs, C9 inhibited cAMP accumulation through CMKLR1. CMKLR1 is known to activate the G_i_ class of G proteins,^9^ suggesting that C9 is probably activating G_i_, and thereby inhibits adenylyl cyclase. This mechanistic study is the first to identify the signaling pathway linking C9 with contraction and is consistent with other G_i_‐coupled vasoconstrictors acting on, for example, α_2_‐adrenoceptors in the peripheral circulation^39^ or M_2_ muscarinic receptors on smooth muscle.^40^

C9‐evoked contraction translated into an increase in MAP in rats in vivo, which was attenuated by CCX832. This confirms that CMKLR1 has a role in blood pressure regulation, matching its expression in the medial layer of rat vessels.^19^ CCX832 did not completely abrogate the pressor response to C9, and this is thought to be because it is a competitive antagonist and therefore its effects are expected to be surmountable. It is likely that CCX832 was not at a high enough concentration to completely block the increase in blood pressure, and because of the compound's solubility, it was not possible to increase the concentration further. Experiments in vitro suggest that inhibition of cAMP accumulation contributes to the contraction evoked by C9 in rat aorta, consistent with our results in human cells. Our in vitro and in vivo studies in rats have therefore identified a robust animal model for further studies into the vasculature actions of chemerin and CMKLR1. Importantly, these direct actions of chemerin are in agreement with previous in vitro studies in rat aorta.^19^ Indirect actions have also been reported in rat aorta, where chemerin modulates the actions of other vasoactive mediators.^41^, ^42^ A previous study by Kunimoto et al^20^ identified that chronic infusion of chemerin caused an increase in systolic blood pressure in mouse in vivo, but they did not identify the mechanism. Watts et al^19^ reported that chemerin had no constrictor action in mouse aorta, even though CMKLR1 is present, and this discrepancy suggests that the mouse is an unreliable model and requires further study into how chemerin modulates blood pressure in mice.

### Biased Agonism at CMKLR1

Biased agonism is emerging as an important concept in GPCR research and has the potential to revolutionize how novel drugs for cardiovascular disease treatment are made.^43^, ^44^ In this study, we used chemerin(21–157) as our reference compound because it is found in human ascitic fluid^9^ and is reported to be the most active endogenous isoform of chemerin.^1^ We have identified that C‐terminal fragment, C9, previously reported to mimic the actions of chemerin,^18^ has a similar potency to chemerin in cAMP inhibition assays only, but is significantly less potent at activating β‐arrestin. We saw a similar phenomenon with C‐terminal fragment C13, suggesting a trend for shorter C‐terminal fragments of chemerin to be strongly biased toward activation of G_i_ protein pathway over the β‐arrestin pathway at the CMKLR1 receptor. This suggests that endogenous chemerin fragments have the potential to exhibit a spectrum of activities at different signaling pathways. Given that there are reported to be many cleavage sites within the protein^36^, ^45^ and it is not yet known what the active form of chemerin is in the vasculature, this could be important, and others in the field have already noted potential at CMKLR1.^46^ Chemerin has a reverse chemokine conformation^18^ and chemokines bind to their receptors in a 2‐site model, where the C‐terminal core of the chemokine is necessary for binding and the flexible N‐terminus for activation.^47^ This therefore suggests that chemerin could activate its receptor in a similar way, with the N‐terminus and C‐terminus being able to stabilize different conformations of the receptor and therefore activate different signaling pathways.

### Chemerin/CMKLR1 Contribution to Cardiovascular Disease?

**Figure 9:**
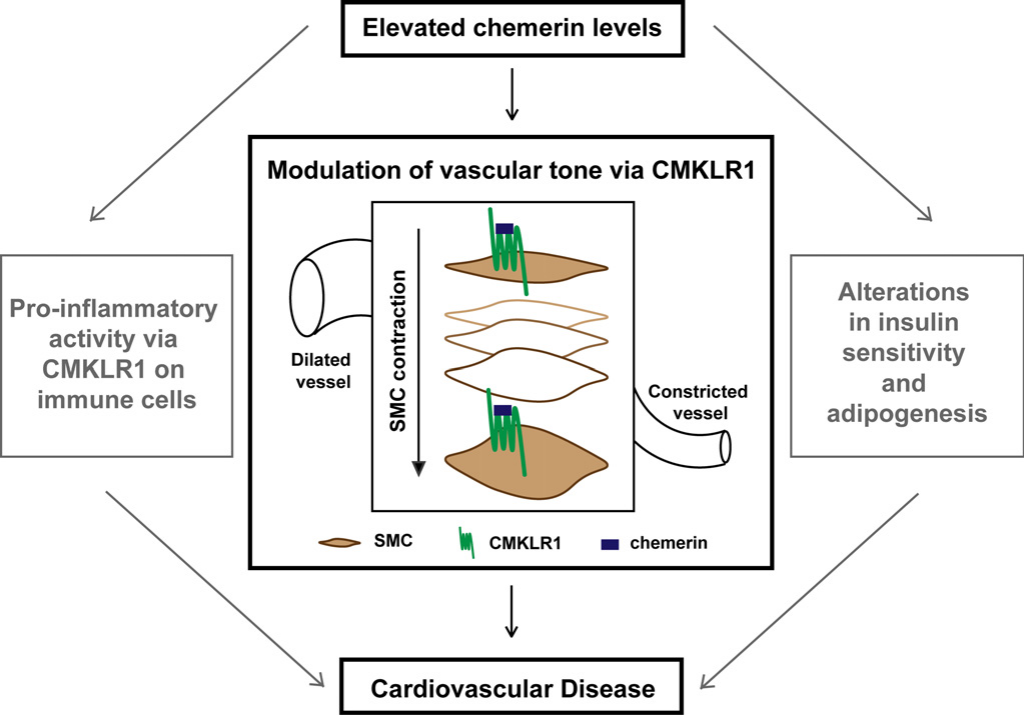
Chemerin is a multifaceted protein that is emerging as a potential contributor to cardiovascular disease. This study identifies a novel role of chemerin in modulation of vascular tone in humans. CMKLR1 indicates chemokine‐like receptor 1; SMC, smooth muscle cells.

Activation of CMKLR1 by chemerin has well‐established roles in inflammation, adipogenesis, and insulin sensitivity, and thus chemerin has a firm role in the pathology of obesity and metabolic syndrome.^1^ There is increasing recognition of the association between metabolic syndrome and cardiovascular disease. High blood pressure could be a potential link given that it contributes both to metabolic syndrome and is a major risk factor for cardiovascular disease. Circulating chemerin concentration positively correlates with both systolic and diastolic blood pressure in a range of patient populations, particularly those with obesity,^48^ metabolic syndrome,^49^, ^50^, ^51^, ^52^ type 2 diabetes mellitus,^53^, ^54^ and type 2 diabetes mellitus with hypertension,^7^ and these patient groups are at higher risk of developing cardiovascular disease. Our data show, for the first time, that chemerin causes concentration‐dependent contraction of human blood vessels and has a direct effect on blood pressure in rats by binding to CMKLR1 on the smooth muscle layer. This allows us to propose a novel mechanism by which chemerin contributes to both cardiovascular disease and metabolic syndrome in addition to its well‐established roles (Figure 9).

## Conclusions

We provide evidence that the G‐protein‐coupled receptor, CMKLR1 which is activated by chemerin, is a tractable drug target. This study identifies a novel role of CMKLR1 in contraction of human resistance arteries. The C‐terminal nonapeptide, C9, has been identified as a potent agonist, and CCX832 is shown to be a highly selective antagonist of CMKLR1, both of which are effective in vitro and in vivo in rat models. Importantly, our study has conclusively identified that CCX832 blocks the constrictor effects of chemerin, providing a proof of principle that targeting the chemerin/CMKLR1 axis could deliver a novel therapeutic opportunity in the treatment of hypertension.

## Sources of Funding

This work was supported by the British Heart Foundation (FS/12/64/30001 [to AJK], FS/14/59/31282 [to CR], and PG/09/050/27734); Wellcome Trust (100780/Z/12/Z [to LY], 101844 [to CWT], 107715/Z/15/Z [to APD and JJM], and 096822/Z/11/Z [to APD and PY]); the Raymond and Beverley Sackler Fellowship (to LY), and the Medical Research Council (MRC MC_PC_14116; to APD) and by the Pulmonary Hypertension Association and the Cambridge Biomedical Research Centre. Biomedical Resources (grant 099156/Z/12/Z).

## Disclosures

None.
